# Tetrahydroxystilbene Glucoside Attenuates Neuroinflammation through the Inhibition of Microglia Activation

**DOI:** 10.1155/2013/680545

**Published:** 2013-11-14

**Authors:** Feng Zhang, Yan-Ying Wang, Jun Yang, Yuan-Fu Lu, Jie Liu, Jing-Shan Shi

**Affiliations:** ^1^Department of Pharmacology and Key Laboratory of Basic Pharmacology of Guizhou Province, Zunyi Medical University, Zunyi, Guizhou 563099, China; ^2^Pharmacy School, Zunyi Medical University, Zunyi, Guizhou 563099, China

## Abstract

Neuroinflammation is closely implicated in the pathogenesis of neurological diseases. The hallmark of neuroinflammation is the microglia activation. Upon activation, microglia are capable of producing various proinflammatory factors and the accumulation of these factors contribute to the neuronal damage. Therefore, inhibition of microglia-mediated neuroinflammation might hold potential therapy for neurological disorders. 2,3,5,4′-Tetrahydroxystilbene-2-O-**β**-D-glucoside (TSG), an active component extracted from *Polygonum multiflorum*, is reported to be beneficial for human health with a great number of pharmacological properties including antioxidant, free radical-scavenging, anti-inflammation, antilipemia, and cardioprotective effects. Recently, TSG-mediated neuroprotective effects have been well demonstrated. However, the neuroprotective actions of TSG on microglia-induced neuroinflammation are not known. In the present study, microglia BV2 cell lines were applied to investigate the anti-neuroinflammatory effects of TSG. Results showed that TSG reduced LPS-induced microglia-derived release of proinflammatory factors such as TNF**α**, IL-1**β**, and NO. Moreover, TSG attenuated LPS-induced NADPH oxidase activation and subsequent reactive oxygen species (ROS) production. Further studies indicated that TSG inhibited LPS-induced NF-**κ**B signaling pathway activation. Together, TSG exerted neuroprotection against microglia-mediated neuroinflammation, suggesting that TSG might present a promising benefit for neurological disorders treatment.

## 1. Introduction

Central nervous system (CNS) is characterized by an immunologically privileged site due to lack of lymphatic infiltration and the limited inflammatory capacity with the presence of blood-brain barrier [1]. Recently, neuroinflammation has been increasingly implicated in the pathogenesis of neurological disorders, including trauma, stroke, brain infections, ischemia, and neurodegenerative diseases, such as Alzheimer's disease (AD), Parkinson's disease (PD), Huntington's disease (HD), multiple sclerosis, and amyotrophic lateral sclerosis [2]. The hallmark of neuroinflammation is considered to be the activation of glial cells, particular microglia [3]. Microglia are the primary immune cells resident within the CNS and serve as the first line of defense to maintain homeostasis during brain injury or disease occurrence [4]. A wide range of stimuli disrupts brain physiological homeostasis and triggers microglia activation. Once activated, microglia are capable of producing a large number of proinflammatory cytokines such as tumour necrosis factor *α* (TNF*α*), interleukin 1*β* (IL-1*β*), nitric oxide (NO), and reactive oxygen species (ROS). The release and accumulation of these microglia-derived proinflammatory factors are thought to enhance the neuronal damage, especially in neurodegenerative diseases [5]. However, the damaged neurons secrete the neurotoxic soluble factors and, in turn, induce microglia reactivation [6]. Taken together, a vicious cycle causing the prolonged neuroinflammation and the progressive neurodegeneration is created [7]. Thus, the inhibition of microglia activation probably possesses a potential therapy for neuroinflammation-related neurological disorders. 

The 2,3,5,4′-tetrahydroxystilbene-2-O-*β*-D-glucoside (TSG), an active component extracted from the dried tuber root of *Polygonum multiflorum*, has been reported to be beneficial for human health and used as an antiaging agent [8]. Recent studies have shown that TSG presents a great number of pharmacological properties including antioxidant, free radical-scavenging, anti-inflammation, antilipemia, and cardioprotective effects [9]. Moreover, TSG-mediated neuroprotective effects have been well demonstrated. TSG exhibits a significant neuroprotection against ischemic brain injury, in *in vitro* and *in vivo* studies [10]. In amyloid-*β*_(1-42)_-induced learning and memory deficits rat model, TSG exerts anti-AD properties through the protection of synaptic structure and function [11]. Also, TSG could antagonize age-related *α*-synuclein overexpression in the hippocampus of APP transgenic AD mouse model [12]. However, the neuroprotective actions of TSG on neuroinflammation, particularly microglial activation, are not known. 

In the present study, microglia BV2 cell lines were applied to investigate the anti-neuroinflammatory effects of TSG on lipopolysaccharide- (LPS-) induced microglial activation and further elucidate the possible mechanisms underlying TSG-mediated neuroprotective properties.

## 2. Material and Methods

### 2.1. Reagents

TSG was obtained from the National Institute for the Control of Pharmaceutical and Biological Products (Beijing, China). LPS (*Escherichia coli* strain O111:B4) and the fluorescence probe dichlorodihydrofluorescein diacetate (DCFH-DA) were purchased from Calbiochem (San Diego, CA, USA). Superoxide dismutase (SOD) and MTT were available from Sigma-Aldrich (St. Louis, MO, USA). WST-1 was purchased from Dojindo Laboratories (Gaithersburg, MD, USA). SYBR green polymerase chain reaction (PCR) master mix and RNeasy Kits were purchased from Applied Biosystems (Cheshire, UK) and Qiagen (Valencia, CA, USA), respectively. Trizol reagent and the materials for cell cultures were obtained from Invitrogen (Carlsbad, CA, USA). Anti-gp91 and anti-p47 antibodies were purchased from BD Transduction Laboratories (San Jose, CA, USA) and Upstate Biotechnology Inc. (Lake Placid, NY, USA), respectively. Anti-CD11b antibody was obtained from Abcam Inc. (Cambridge, MA, USA). Other primary antibodies were the products of Cell Signaling Technology (Beverly, MA, USA). 

### 2.2. Cell Cultures

Mouse microglial BV2 cell lines were obtained from the Cell Culture Center, Institute of Basic Medical Sciences, Chinese Academy of Medical Sciences (Beijing, China). The cultures were maintained in Dulbecco's Modified Eagle Medium/F12 (DMEM/F12) medium supplemented with 10% heat-inactivated fetal bovine serum (FBS), 100 U/mL penicillin, and 100 *μ*g/mL streptomycin at 37°C in the humidified atmosphere of 5% CO_2_ and 95% air. At the treatment time, cell cultures were changed to the treatment medium containing 2% FBS, 100 U/mL penicillin, and 100 *μ*g/mL streptomycin. 

### 2.3. MTT Assay

Cell viability was evaluated by MTT assay. Briefly, cells were dissociated and seeded at 1 × 10^5^/well in 96-well plates. After 24 h incubation, cells were treated with various concentrations of TSG (20–80 *μ*M) with or without LPS (1 *μ*g/mL) treatment for an additional 24 h followed by the incubation of MTT solution (0.5 mg/mL) at 37°C for 4 h. Then, the culture medium was removed, and cells were lysed with 200 *μ*L DMSO and shaken for 15 min. The optical density values of the solubilized formazan products in each well were measured at 570 nm using an automated microplate reader. 

### 2.4. Real-Time RT-PCR Assay

Total RNA was extracted by Trizol reagent and purified with RNeasy Kits. The PCR primers were designed using ABI Primer Express software (Applied Biosystems, Foster City, CA, USA). The primer sequences were TNF*α*: TCGTAGCAAACCACCAAGCA (F), CCCTTGAAGAGAACCTGGGAGTA (R); IL-1*β*: CACCTCTCAAGCAGAGCACAGA (F), GGGTTCCATGGTGAAGTCAACT (R); inducible nitric oxide synthase (iNOS): GTGCTAATGCGGAAGGTCATG (F), CGCTTCCGACTTTCCTGTCT (R), and *β*-actin: TCCTCCTGAGCGCAAGTACTCT (F), GCTCAGTAACAGTCCGCCTAGAA (R). Total RNA was reversely transcribed by MuLV reverse transcriptase and Oligo-dT primers. The SYBR green PCR Master Mix was performed for real-time PCR analysis. The relative mRNA expression difference among groups was measured via cycle time (Ct) values normalized with *β*-actin of the same sample. The relative mRNA expressions in each group were performed and calculated through setting LPS-treated group at 100%. 

### 2.5. TNF*α*, IL-1*β*, and Nitrite Assay

The levels of TNF*α* and IL-1*β* released from cells in the culture supernatants were measured by the enzyme-linked immunosorbent assay (ELISA) kits from R&D Systems (Minneapolis, MN, USA). The NO production was evaluated by detecting the accumulated content of nitrite in the culture medium with the Griess reagent. Briefly, cells were seeded at 1 × 10^6^/well in 24-well plates and treated with TSG with or without LPS. After drug treatment for 24 h, the culture medium was harvested and mixed with an equal volume of Griess reagent (0.1% N-(1-naphthyl) ethylenediamine dihydrochloride, 1% sulfanilamide and 2.5% H_3_PO_4_). The mixture was incubated in the dark for 10 min at room temperature. The absorbance at 540 nm was determined using a microplate spectrophotometer. The nitrite concentration in the samples was measured based on a sodium nitrite standard curve.

### 2.6. Superoxide Assay

Superoxide production was detected via the SOD-inhibitable reduction of the tetrazolium salt WST-1. Cell cultures in 96-well plates were washed twice with Hank's Balanced Salt Solution (HBSS) without phenol red. Then, cells were incubated with vehicle control and TSG in HBSS at 37°C for 30 min followed by the addition of HBSS with and without SOD (50 U/mL) to each well along with WST-1 (1 mM) in HBSS and LPS. The absorbance at 450 nm was determined through a SpectraMax Plus microplate spectrophotometer every 5 min for 1 h. The different absorbance observed in the presence or absence of SOD was considered to be the amount of superoxide production. 

### 2.7. Intracellular ROS Assay

Intracellular ROS production was measured using the DCFH-DA assay. Cells were seeded in 96-well plates and exposed to DCFH-DA for 1 h followed by TSG pretreatment for 30 min and then treatment with LPS. After incubation at 37°C for an additional 30 min, the fluorescence was detected and read at 485 nm for excitation and 530 nm for emission via a SpectraMax Gemini XS fluorescence microplate reader.

### 2.8. Western Blot Analysis

For the subcellular fractions extraction, cells were lysed in hypotonic lysis buffer and homogenized. Cell lysates were loaded onto a sucrose gradient in lysis buffer and centrifuged at 1600 ×g for 15 min. The supernatant on the sucrose gradient was collected as the cytosolic fractions after centrifugation at 150,000 ×g for 1 h. The pellet was solubilized in hypotonic lysis buffer and collected as the membranous fractions. For the whole cell lysates extraction, cell cultures were washed with cold PBS and lysed with RIPA cell lysis buffer. Cell lysates were incubated on ice for 30 min and then centrifuged at 12,000 ×g for 30 min. The protein levels were quantified by the BCA assay. Membranes were blocked with 5% nonfat milk and then incubated with the following antibodies: anti-CD11b, anti-p47, anti-gp91, anti-phospho-p65, anti-p65, anti-phospho-IKK, anti-IKK, anti-*β*-actin, and horseradish peroxidase (HRP)-conjugated secondary antibodies. The blot films were developed with enhanced ECL reagent.

### 2.9. Statistical Analysis

Data were presented as mean ± SEM from three independent experiments performed in triplicate. Statistical significance was analyzed by one-way ANOVA using GraphPad Prism software (GraphPad Software Inc., San Diego, CA, USA). When ANOVA indicated the significant differences, pairwise comparisons between means were accessed by Bonferroni's *post hoc t*-test with correction. A value of *P* < 0.05 was considered statistically significant.

## 3. Results

### 3.1. TSG Had No Neurotoxicity on BV2 Cells

**Figure 1:**
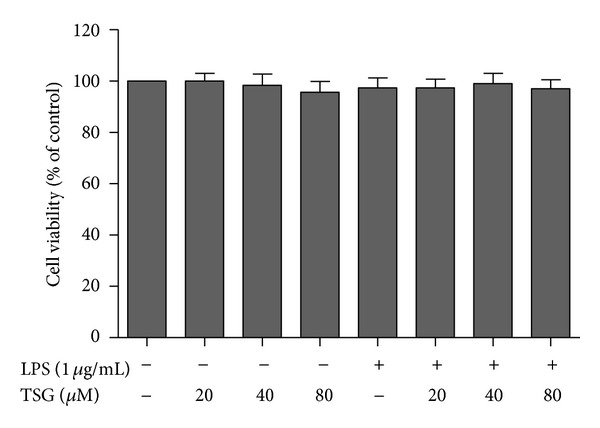
TSG had no neurotoxicity on BV2 cells. Cell viability was determined by MTT assay. Results were expressed as a percentage of the control cultures and were the mean ± SEM from three independent experiments performed in triplicate.

BV2 cells were pretreated with TSG (20–80 *μ*M) for 30 min followed by LPS (1 *μ*g/mL) application for 24 h. MTT assay was performed for the cell viability analysis. As shown in Figure 1, no significant difference among the vehicle control, TSG (20–80 *μ*M) alone, LPS (1 *μ*g/mL), and LPS plus TSG (20–80 *μ*M) treatment was indicated. 

### 3.2. TSG Suppressed LPS-Induced Microglial Activation

**Figure 2:**
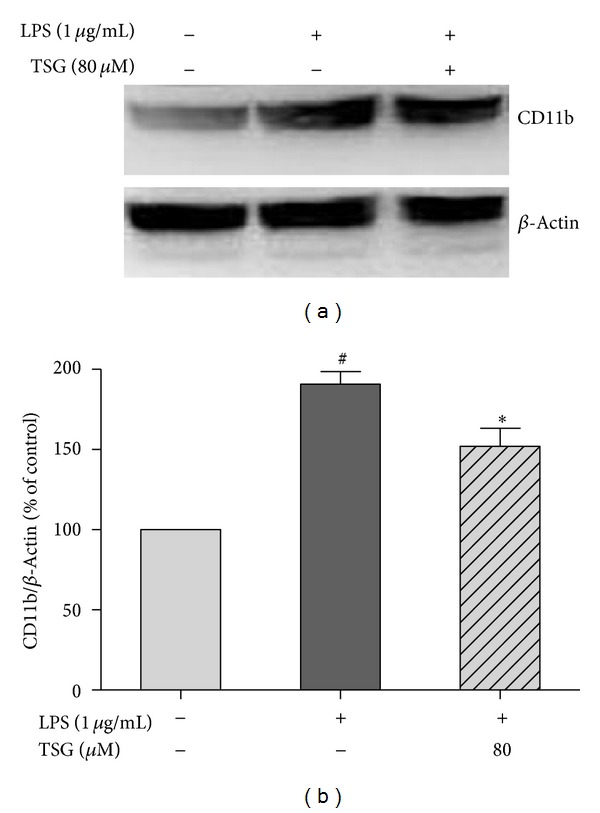
TSG suppressed LPS-induced microglial activation in BV2 cells. The CD11b protein expression was measured by western blotting analysis (a). The ratio of densitometry values of CD11b and *β*-actin was normalized to each respective control group (b). Results were the mean ± SEM from three independent experiments performed in triplicate. ^#^ *P* < 0.05 compared with the control cultures; *P < 0.05 compared with LPS-treated cultures.

BV2 cells were pretreated with TSG (80 *μ*M) for 30 min before the application of LPS (1 *μ*g/mL). Twenty four hours later, the total cell protein was collected and the protein expression of CD11b, the *β*-integrin marker of microglia, which represents microglial activation during neuroinflammation, was detected by western blot assay. As shown in Figures 2(a) and 2(b), TSG pretreatment suppressed LPS-induced microglial activation.

### 3.3. TSG Attenuated LPS-Induced Inflammatory Response in BV2 Cells

**Figure 3:**
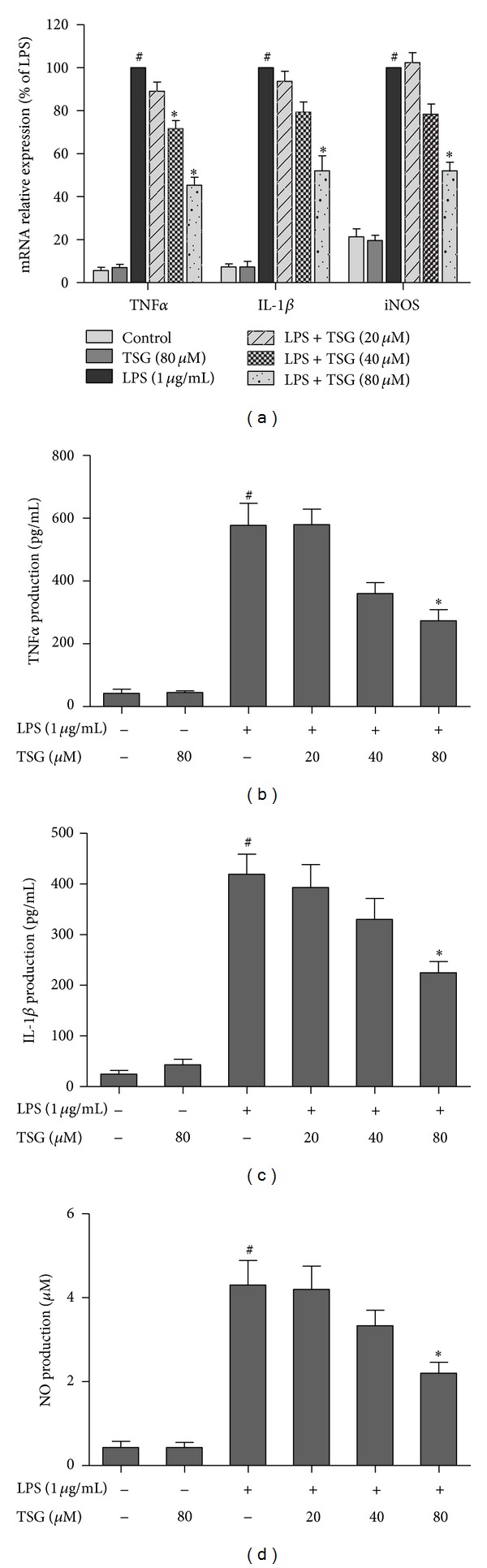
TSG attenuated LPS-induced inflammatory response in BV2 cells. The mRNA expression was determined by real-time RT-PCR (a). The levels of TNF*α* (b), IL-1*β* (c), and NO (d) in BV2 culture medium were detected by ELISA and the Griess reagent, respectively. Results were the mean ± SEM from three independent experiments performed in triplicate. ^#^ *P* < 0.05 compared with the control cultures; *P < 0.05 compared with LPS-treated cultures.

BV2 cells were pretreated with TSG (20–80 *μ*M) for 30 min and then stimulated with LPS (1 *μ*g/mL) for 12 h. The mRNA expressions of proinflammatory factors were measured by real-time RT-PCR. As shown in Figure 3(a), LPS led to an apparent increase in mRNA expressions of TNF*α*, IL-1*β*, and iNOS in BV2 cells. TSG pretreatment significantly inhibited the LPS-induced elevated transcripts of TNF*α*, IL-1*β*, and iNOS. Moreover, after LPS treatment for 24 h, the production of TNF*α*, IL-1*β*, and NO in BV2 culture medium was detected by ELISA and the Griess reagent, respectively. As shown in Figures 3(b), 3(c), and 3(d), pretreatment with TSG significantly decreased LPS-induced production of TNF*α*, IL-1*β*, and NO in BV2 culture medium.

### 3.4. TSG Inhibited LPS-Induced ROS Production and NADPH Oxidase Activation

**Figure 4:**
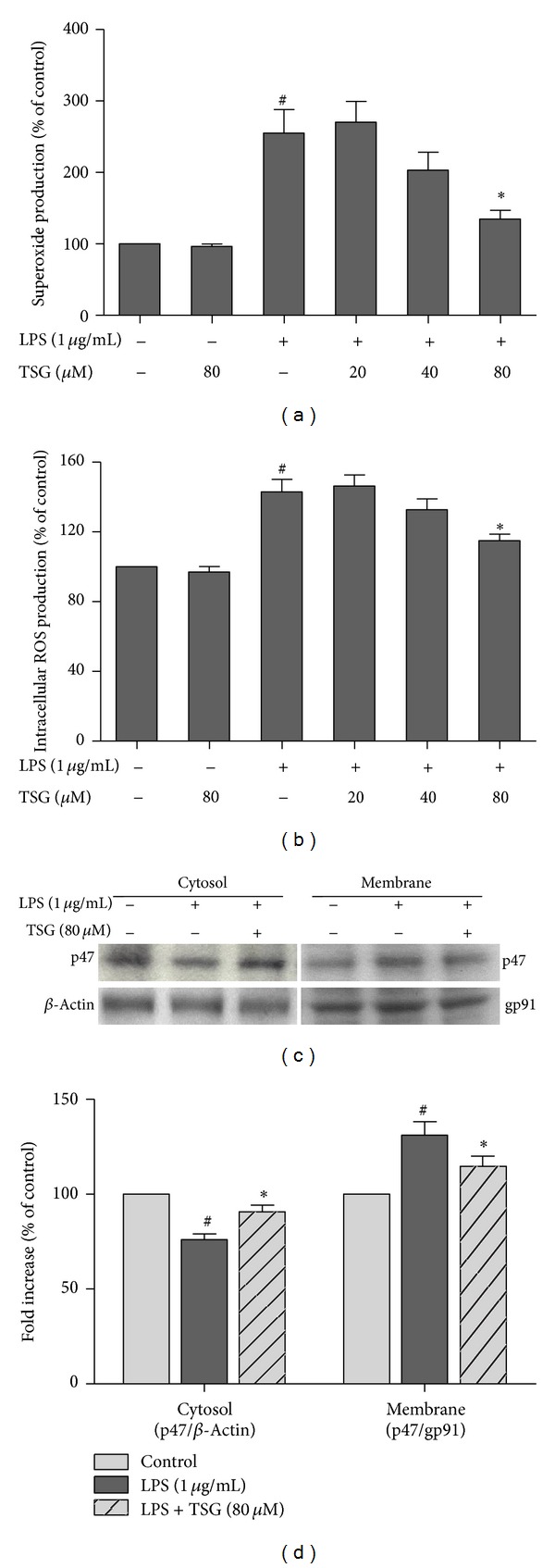
TSG inhibited LPS-induced ROS production and NADPH oxidase activation. The extracellular superoxide level was measured by SOD-inhibitable reduction of WST-1 (a), and the content of intracellular ROS were detected with DCFH-DA (b). The subcellular fractions were isolated, and western blot assay was performed to evaluate the NADPH oxidase subunit p47 levels in the cytosolic and membrane fractions of BV2 cells. *β*-actin and gp91 were applied as the internal cytosolic and membrane controls, respectively, (c). The ratio of densitometry values of cytosolic and membrane p47 compared with *β*-actin and gp91, respectively, was assessed and normalized to each control cultures (d). Results were expressed as a percentage of the control cultures and were the mean ± SEM from three independent experiments performed in triplicate. ^#^ *P* < 0.05 compared with control cultures; *P < 0.05 compared with LPS-treated cultures.

BV2 cells were pretreated with TSG for 30 min before LPS treatment. As shown in Figures 4(a) and 4(b), LPS significantly caused the increased production of extracellular superoxide and intracellular ROS, and this increase could be attenuated by TSG pretreatment. Furthermore, NADPH oxidase activation was investigated by western blot assay after LPS treatment for 6 h. As shown in Figures 4(c) and 4(d), LPS significantly induced the translocation of NADPH oxidase subunit p47 from cytosol to membrane, and TSG pretreatment could ameliorate the LPS-induced increase of p47 translocation.

### 3.5. TSG Suppressed LPS-Induced NF-*κ*B Signaling Pathway Activation

**Figure 5:**
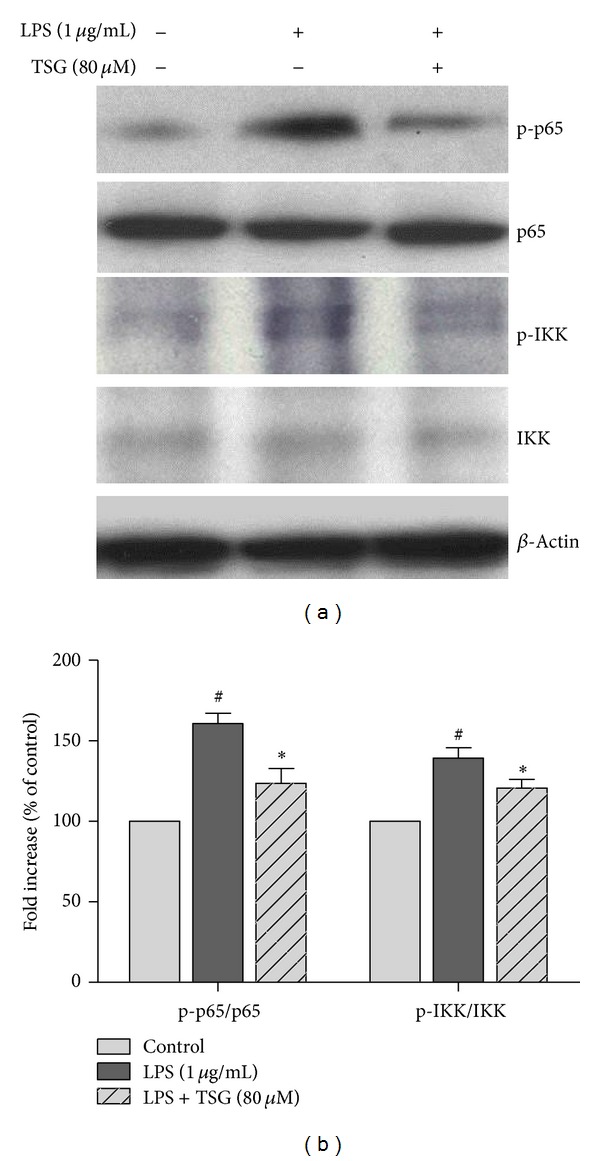
TSG suppressed LPS-induced NF-*κ*B signaling pathway activation. Western blot assay was performed to analyze NF-*κ*B signaling pathway activation (a). The ratio of densitometry values of phosphorylated p65 (p-p65) and IKK (p-IKK) compared with total p65 and IKK, respectively, was analyzed and normalized to each control cultures (b). Results were expressed as a percentage of the control cultures and were the mean ± SEM from three independent experiments performed in triplicate. ^#^ *P* < 0.05 compared with the control cultures; *P < 0.05 compared with LPS-treated cultures.

BV2 cells were pretreated with TSG (80 *μ*M) for 30 min and then incubated with LPS for 6 h. The whole cell lysates were collected, and western blot assay was used for NF-*κ*B signaling pathway activation analysis. As shown in Figures 5(a) and 5(b), LPS significantly elicited phosphorylation of p65 and IKK, and TSG pretreatment reduced LPS-induced NF-*κ*B signaling pathway activation.

## 4. Discussion

The present study indicated that TSG produced neuroprotection against microglia-mediated neuroinflammation. TSG inhibited LPS-induced microglial activation and the proinflammatory factors release. Furthermore, inhibition of NADPH oxidase and NF-*κ*B signaling pathway activation participated in TSG-produced anti-neuroinflammatory effects. 

Increasing evidence has demonstrated that microglial activation and the consequent release of proinflammatory and cytotoxic factors such as TNF*α*, IL-1*β*, NO, and prostaglandin E synthase 2 (PGE_2_) are thought to contribute to the neurodegenerative diseases [13–15]. Analysis of postmortem brains showed that the increased levels of proinflammatory mediators were investigated in patients with neurological disorders [16]. Therefore, inhibition of microglial activation-induced neuroinflammation might be a potential therapeutic strategy for neuroprotection. This study found that TSG inhibited LPS-induced TNF*α*, IL-1*β*, and NO production released by microglia. These results were consistent with the previous studies that TSG suppressed matrix metalloproteinase expression and inflammation in diet-induced atherosclerotic rats [17] and inhibited cyclooxygenase-2 enzyme activity and expression in RAW264.7 macrophage cells [18].

Among the proinflammatory factors released by microglia, ROS play a pivotal role in various neurotoxins-induced neurotoxicity [19, 20]. ROS including superoxide, hydroxyl radicals, peroxy radicals, and hydrogen peroxide are highly reactive, and excessive production of these free radicals leads to lipid peroxidation and DNA damage and, further, induces cell dysfunction and death [21]. Since neurons are fairly sensitive to oxidative stress, oxidative stress damage is believed to contribute to the neuronal loss in neurodegenerative diseases [22]. Several lines of evidence indicate that ROS serve as the secondary messengers to encode and enhance the gene expression of the majority of proinflammatory factors [23]. Importantly, superoxide could react with NO to form highly toxic peroxynitrite to induce neurodegeneration [24]. Furthermore, NADPH oxidase is recognized as the key ROS-producing enzyme during inflammation and is widely expressed in various immune cells such as macrophages, eosinophils, microglia, and neutrophils [25]. Upon NADPH oxidase activation, the cytosolic subunits (p40, p47, p67, and Rac1) translocate to the membrane-binding cytochrome b558 consisting of p22 and the catalytic subunit gp91 to assemble the functional oxidase and catalyze the reduction of oxygen to superoxide free radical [26]. Numerous studies have indicated that the pharmacological inhibition and the genetic deletion of NADPH oxidase protects against LPS, rotenone, paraquat, and 1-methyl-4-phenyl-1,2,3,6-tetrahydropyridine (MPTP)-induced neurodegeneration [27]. Thus, NADPH oxidase might be a potential therapeutic target for neuroinflammation-related neurological disorders treatment. Recent studies have reported that TSG protects against 6-OHDA-induced apoptosis in PC12 cells through its antioxidative stress actions [28]. This study implied that TSG produced neuroprotection through the inhibition of the translocation of p47 from cytosol to membrane and the subsequent ROS production in microglia. 

This study also found that TSG suppressed LPS-induced microglia NF-*κ*B signaling pathway activation. It has been strongly suggested that NF-*κ*B signaling pathway is an important regulator of neuroinflammation [29]. Optimal NF-*κ*B activation requires phosphorylation of the NF-*κ*B subunit p65 protein by IKK along with the activation of a variety of NF-*κ*B inhibitors I*κ*Bs [30]. Furthermore, NF-*κ*B activation is discerned in the brains of patients with neurodegenerative diseases and animal models of neurological disorders [31]. Interestingly, the marked colocalization of p65 with CD11b-labeled activated microglia in the substantial nigra of postmortem PD patients is particularly indicated [32]. Therefore, inhibition of NF-*κ*B activation suppressed microglia activation and the consequent release of proinflammatory factors and were eventually beneficial for neuroprotection [33]. In addition, increasing evidence demonstrated that activated microglia NADPH oxidase transfers electrons across the plasma membrane from NADPH to molecular oxygen and then induces ROS production. These free radicals not only are neurotoxic to neurons but also, in turn, enter microglia to induce the downstream signaling pathways such as NF-*κ*B cascade pathway activation in microglia-mediated neuroinflammation [34, 35]. On the other hand, inhibition of NADPH oxidase and ROS scavenging ameliorates the activation of NF-*κ*B pathway, suggesting that there is a crosstalk between NADPH oxidase-generating ROS and NF-*κ*B pathway [36]. Here, TSG-inhibited NF-*κ*B pathway activation would likely result from the inhibition of NADPH oxidase activity and the subsequent ROS production.

In conclusion, this study demonstrated that TSG mediated neuroinflammation through the inhibition of microglial activation and the subsequent release of proinflammatory factors. These neuroprotective effects might be closely associated with the attenuated activation of NADPH oxidase and NF-*κ*B signaling pathways. This study extends the anti-inflammatory activities of TSG, implicating that TSG might become a promising candidate for the treatment of neuroinflammation-induced neurological disorders.
